# Kidney Cancer Incidence and Mortality Disparities Involving American Indians/Alaska Natives: An Analysis of the Oklahoma Central Cancer Registry (OCCR)

**DOI:** 10.1155/2022/2689386

**Published:** 2022-06-19

**Authors:** Victoria Gonzalez, Michael Suflita, Amanda Janitz, Janis Campbell, Andrew G. McIntosh, Kelly Stratton, Michael S. Cookson, Daniel C. Parker

**Affiliations:** ^1^Department of Urology, The University of Oklahoma Health Sciences Center & The Stephenson Cancer Center, Oklahoma City, Oklahoma, USA; ^2^Department of Biostatistics & Epidemiology, The University of Oklahoma Health Sciences Center, Oklahoma City, Oklahoma, USA

## Abstract

**Purpose:**

This cohort study describes the differences in kidney cancer age-adjusted incidence and mortality rates between American Indians/Alaskan Natives (AI/ANs) and Whites in Oklahoma. Additionally, rates for the U.S. are updated to establish an epidemiological comparison between Oklahoma and the rest of the country.

**Materials and Methods:**

Kidney cancer age-adjusted incidence and mortality rates for Oklahoma were gathered using the Oklahoma Central Cancer Registry since 1999. National rates were obtained from the Center for Disease Control and Prevention Wide-ranging Online Data for Epidemiologic Research database between 1997 and 2017. Rate ratios were used to compare incidence and mortality rates for AI/ANs and Whites within Oklahoma as well as the entire country. Joinpoint regression models were created to illustrate trends in kidney cancer incidence and mortality.

**Results:**

The age-adjusted incidence rate of kidney cancer in Oklahoma for AI/ANs and Whites was 32.3 and 15.8 per 100,000, respectively, for an incidence rate ratio of 2.04. The national incidence rate ratio was 0.89. The age-adjusted mortality rate in Oklahoma for AI/ANs and Whites was 9.78 and 4.98 per 100,000, respectively, for a mortality rate ratio of 1.98. Oklahomans, irrespective of race, fare worse in terms of kidney cancer mortality compared to the rest of the country.

**Conclusions:**

In Oklahoma, AI/ANs are more likely than Whites to have a kidney cancer diagnosis. AI/ANs are twice as likely to die from kidney cancer than Whites in Oklahoma. AI/AN populations in certain states may benefit from kidney cancer early screening initiatives.

## 1. Introduction

Kidney cancer is the 8^th^ most commonly diagnosed cancer in the United States and is responsible for nearly 2.5% of all cancer deaths each year [1]. Clear disparities exist in the distribution of the disease across racial populations in the U.S. [2, 3]. For example, among American Indians and Alaskan Natives (AI/AN), kidney cancer is the 4^th^ most common cancer (35.7 per 100,000) [2] and the 5^th^ worst cancer for mortality (6.1 deaths per 100,000) [3]. Additionally, a recent study showed that kidney cancer incidence in AI/ANs was elevated compared to Whites in almost all regions of the U.S. [4]. While national rates of kidney cancer diagnosis have been decreasing overall in the US [5], data for AI/ANs show significant increases in the incidence of kidney cancer by as much as 2.4% per year since 1999 [2]. A previous study that examined epidemiological trends of all cancer in Oklahoma between 2005 and 2009 using a local registry found that AI/ANs had an age-adjusted kidney cancer incidence rate (AAIR) of 30.8 per 100,000 which was nearly double that for the White population (16.2 per 100,000) [6]. However, a comparison of mortality rates from kidney cancer in Oklahoma between AI/ANs and Whites has yet to be reported.

Oklahoma ranks second in the U.S. for the largest AI/AN population (482,760 people in 2010) [7]. Due to the structure of the Surveillance, Epidemiology, and End Results (SEER) program, only AI/ANs from Arizona, Alaska, and the Cherokee Nation participate in the national database [8, 9]. The Cherokee Nation makes up approximately 40 percent of the AI/AN population in Oklahoma [7]. The Oklahoma Central Cancer Registry (OCCR) database, however, includes tribal health facilities which represent all 39 tribes in Oklahoma. This creates an opportunity to study the kidney cancer incidence and mortality rate disparities in greater detail by utilizing state and local registries that capture data from all AI/AN tribes. For example, when kidney cancer mortality trends from the National Cancer Database (NCDB) were compared with a statewide registry from Arizona, a 33% increased risk of kidney cancer mortality for AI/ANs in that state was demonstrated which was not evident in the NCDB [10].

The purpose of this study is to define kidney cancer incidence and mortality rate disparities between AI/ANs and Whites in Oklahoma. Additionally, the study intends to update the national epidemiological rates and to identify temporal trends that may illustrate a better portrait of the kidney cancer burden in the AI/AN population. By obtaining more insight into racial disparities that exist for kidney cancer in Oklahoma, further efforts can be made towards the development of quality improvement projects that are generalizable to the rest of the nation's AI/AN population.

## 2. Materials and Methods

Oklahoma kidney cancer rates were obtained from OK2SHARE (https://www.health.state.ok.us/), which is a freely accessible web portal from of the Oklahoma State Department of Health (OSDH). The cancer statistics from this portal include all cancers diagnosed and treated in Oklahoma since January 1, 1997, as recorded by the Oklahoma Central Cancer Registry (OCCR). The registry is a member of the North American Association of Central Cancer Registries and has been awarded gold status for data completeness [11]. OCCR utilizes SEER site groups for primary kidney tumor diagnoses based on the International Classification of Diseases of Oncology, Third Edition/World Health Organization (ICDO-3/WHO), which included C64.9 and C65.9 but excluded histology codes M-9050:9055, 9140, 9590:9989.

Incidence and mortality data were gathered from OK2SHARE for all available years (incidence: 1999-2018 and mortality: 1999-2015) for the two races: White and American Indian/Alaskan Native. Data were then stratified by gender, race, cancer stage, year of diagnosis, and age. Indian Health Service (IHS) racial categories were used to decrease misclassification based on race [12], and only IHS-corrected race data is utilized in OCCR. To prevent exclusion of AI/ANs, the Oklahoma IHS patient registration database is linked to the cancer registry to identify AI/AN cases that have been misclassified. Since IHS facilities are prominent in Oklahoma and IHS eligibility is limited to those that fit the individual tribal requirements, the use of these corrected categories represents the AI/AN population more accurately. The age groups were divided into categorical variables (<50, 50-69, and >70). The cancer stages were divided into four categorical variables (localized, regional, distant, and unknown). Date of diagnosis was divided into five-year intervals (1999-2003, 2004-2008, 2009-2013, and 2014-2018).

Data for United States cancer rates were obtained from the Center for Disease Control and Prevention Wide-ranging Online Data for Epidemiologic Research (CDC WONDER) database. Both incidence and mortality data were collected from 1999 to 2017. All data pertaining to Oklahoma was excluded from the CDC WONDER database query in this study in order to eliminate counting Oklahoma data twice. Similar to the Oklahoma data, stratifications were created based on gender, race (using IHS race categories), cancer stage, year of diagnosis, and age.

To calculate the cancer incidence rates, the 2010 U.S. Census was used as a population estimate from 1999 to 2017. The 2000 U.S. standard population was used to calculate the AAIR and age-adjusted mortality rates (AAMR) [13]. All rates were calculated per 100,000 people. The 95% confidence intervals were calculated by the methods described by Fay and Feuer [14]. Rate ratios for AI/ANs were calculated using White rates for comparison. Using the methods described by Agresti [15], the 95% confidence intervals were calculated for the rate ratios (RR). The RR was used to compare the incidence and mortality of kidney cancer in AI/ANs to Whites overall and by gender.

Joinpoint regression models were created in order to examine temporal trends in kidney cancer incidence and mortality. This analysis was used to compare the time trends of the incidence rates and the mortality rates from 1997 to 2017 by race and gender. The models were fit to a maximum of three joinpoints. Additionally, the annual percent change (APC) was calculated for the population subgroups. These analyses were completed using Joinpoint Regression Program Version 4.8.0.1 in similar fashion to previously published epidemiological studies of oncologic racial disparities in Oklahoma [16].

## 3. Results

### 3.1. Participant Demographic Characteristics

Nationally, there were 6,542 AI/AN and 809,816 White cancer cases diagnosed from 1999 to 2017 (Table 1). Oklahoma had 1,938 AI/AN and 11,996 White kidney cancer cases from 1997 to 2018. In the U.S. during the study period, 1,507 AI/AN and 213,033 Whites died from kidney cancer. For mortality rates, OCCR contained data from 408 AI/ANs and 2,876 Whites. AI/ANs and Whites were well matched for analysis in terms of proportions of each gender represented, age at diagnosis, and clinical stage at diagnosis (for Oklahoma incidence data only). The only significant difference between the two racial groups concerned the years of diagnosis (*P* = 0.03) and mortality (*P* < 0.01) on a national level, for which AI/ANs had a higher proportion of both categories represented in more recent years.

### 3.2. Incidence and Mortality Rate Ratios

The overall AAIR of kidney cancer in Oklahoma for AI/ANs and Whites was 32.3 (95% CI 32.1-33.5) and 15.8 (95% CI 15.5-16.1) per 100,000, respectively (Table 2). This corresponds to an overall incidence rate ratio of 2.04 (95% CI 1.96-2.13). On the national level, the AAIRs were 14.0 (95% CI 13.7-14.3) and 15.7 (95% CI 15.7-15.7) per 100,000 for AI/ANs and Whites, respectively. The rate ratio for kidney cancer incidence derived from the U.S. data was 0.89 (95% CI 0.87-0.91). These data suggest that AI/ANs in Oklahoma have more than twice the AAIR of kidney cancer compared to Oklahoma Whites as well as compared to AI/ANs elsewhere in the country. In Oklahoma, this trend of doubling the incidence rate of kidney cancer for AI/ANs compared to Whites held true also across gender categories.

A similar pattern emerged from the kidney cancer AAMR data. In Oklahoma, the mortality rate from kidney cancer among AI/ANs and Whites was 9.7 (95% CI 8.9-10.5) and 4.9 (95% CI 4.7-5.1) per 100,000, respectively. This corresponds to a mortality rate ratio of 1.98 (95% CI 1.81-2.17). This finding also held true across gender categories with AI/AN males and females dying at 1.97 (95% CI 1.77-2.19, 95% CI 1.68-2.30) times the rate as their White counterparts. However, nationally there appeared to be no significant difference in the AAMR of AI/ANs (3.9 per 100,000) compared with Whites (4.0 per 100,000) with a rate ratio 0.98 (95% CI 0.94-1.01).

### 3.3. Joint Point Regression Results

**Figure 1:**
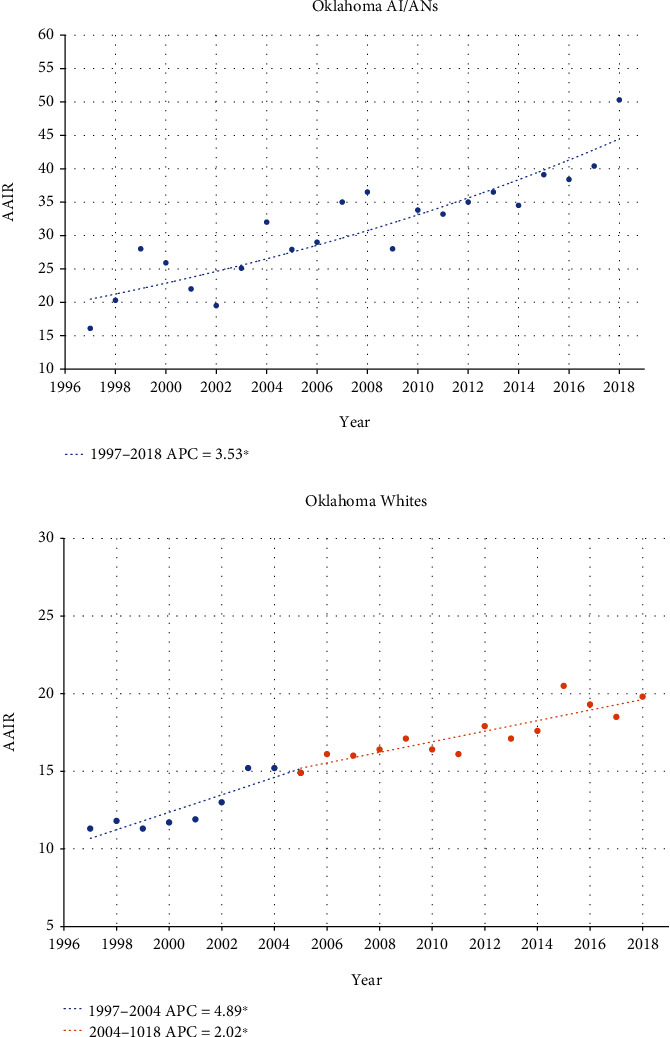
Joinpoint regression comparing AAIR between Oklahoma AI/ANs and Whites over time (rates per 100,000 population). ^∗^Indicates that the annual percentage change (APC) is significantly different from zero at the alpha = 0.05 level.

**Figure 2:**
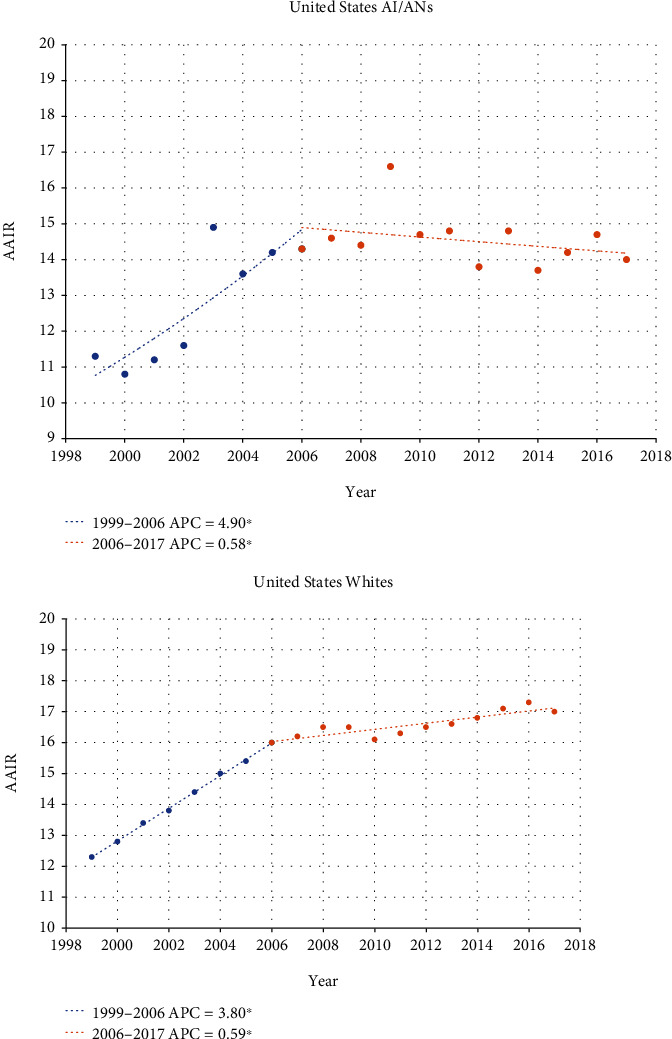
Joinpoint regression comparing AAIR between United States AI/ANs and Whites over time (rates per 100,000 population). ^∗^Indicates that the annual percentage change (APC) is significantly different from zero at the alpha = 0.05 level.

In Oklahoma, the AAIR of kidney cancer among AI/ANs has been increasing from 1999, with an overall annual percentage change of 3.53 and no inflection points (Figure 1). Whites, on the other hand, had an increasing rate of kidney cancer incidence at an APC of 4.89 until 2004, at which time the incidence rate slowed to an APC of 2.02. This suggests that since 2004, the incidence of kidney cancer in AI/ANs has been increasing at an APC of 1.51% faster than Whites. This is much different than the U.S. national incidence rate data (Figure 2). In the U.S. between 1999 and 2006, both AI/ANs and Whites had similarly rising rates of kidney cancer diagnosis (AI/AN APC 4.90 and White APC 3.80). However, in 2007, the incidence rate of kidney cancer among AI/ANs began to fall at an APC of -0.58. This similar inflection towards declining rates of diagnosis was not seen in Whites, although their rate of increase drastically slowed to an APC of 0.59.

**Figure 3:**
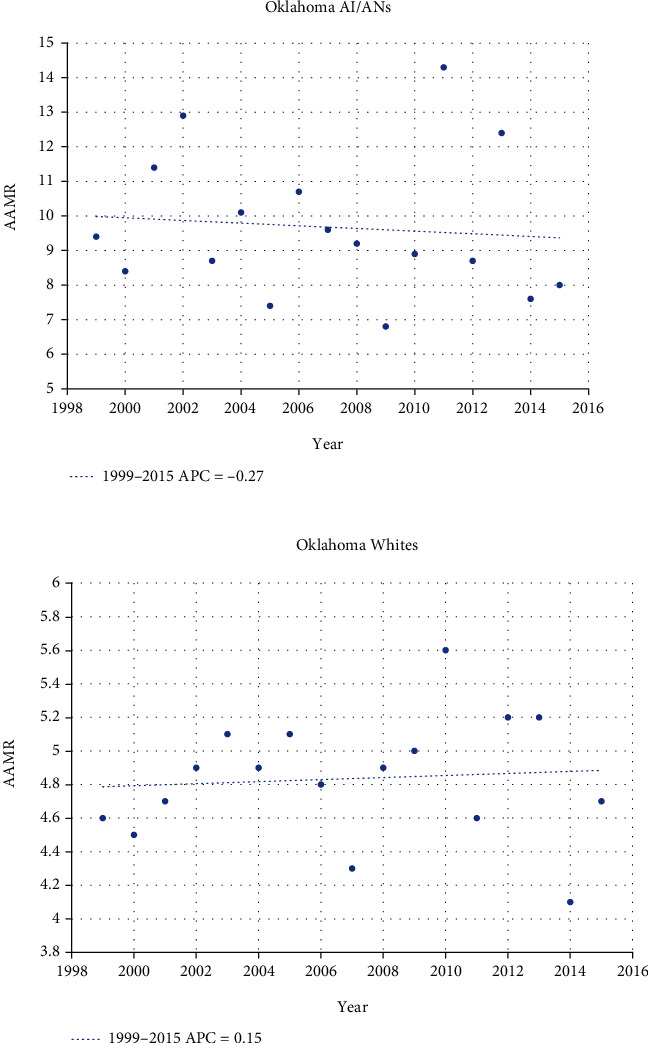
Joinpoint regression comparing AAMR between Oklahoma AI/ANs and Whites over time (rates per 100,000 population). ^∗^Indicates that the annual percentage change (APC) is significantly different from zero at the alpha = 0.05 level.

**Figure 4:**
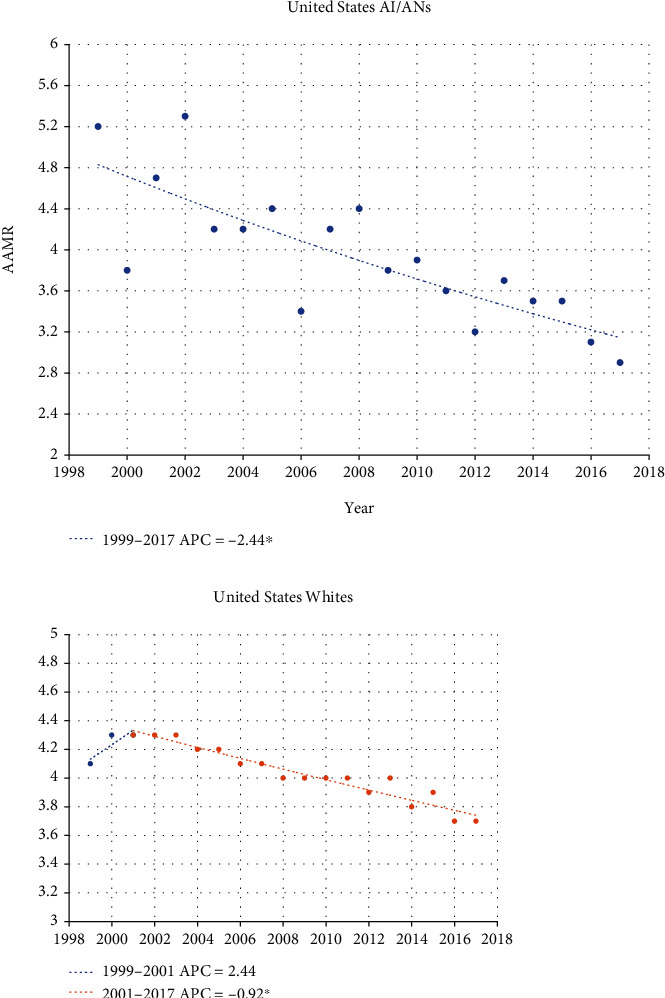
Joinpoint regression comparing AAMR between United States AI/ANs and Whites over time (rates per 100,000 population). ^∗^Indicates that the annual percentage change (APC) is significantly different from zero at the alpha = 0.05 level.

Mortality rates from kidney cancer for both AI/ANs and Whites have been steadier. AI/ANs in Oklahoma experienced a slight decrease in mortality rate over the study period, with an APC of -0.27 (Figure 3). Whites, however, have had a slowly *increasing* mortality rate with an APC of 0.15. No inflection points towards shifting rates have been seen for either race category in Oklahoma in terms of mortality. Certainly, the national data shows that Oklahomans fare worse in terms of kidney cancer mortality, irrespective of race, compared to the rest of the U.S. For example, despite Oklahoman AI/ANs having a small decrease in mortality rate, the U.S. AI/AN population has seen a much steeper decline in kidney cancer mortality with an APC of -2.44 (Figure 4). While the Oklahoma kidney cancer mortality rate for Whites was slightly increasing over the study period, Whites nationally have seen declining rates of kidney cancer mortality since 2001 at an APC of -0.92.

## 4. Discussion

Our study shows that AI/ANs in Oklahoma are nearly two times more likely than Whites, and also twice as likely as other AI/ANs in the entire nation, to receive a diagnosis of kidney cancer. These data confirm the findings of Campbell et al. who demonstrated an AAIR ratio of 1.90 among the same AI/AN population in Oklahoma compared to Whites between the years of 2005 and 2009 [6]. The current study goes a step farther and reports Oklahoma AAMR ratio data for the first time. In terms of mortality, both AI/AN and White populations in this state fare worse than their counterparts across the U.S.

One explanation for why the Oklahoma racial disparity in terms of incidence and mortality of kidney cancer is more pronounced relative to the U.S. is that previously reported epidemiological studies relying solely on SEER have excluded 60% of this country's second largest AI/AN population. Another explanation, as offered by Valencia and colleagues, is that minority populations may tend to gravitate toward healthcare settings whose epidemiological data are not captured by large databases [10]. Some minority populations tend to use smaller, community-based healthcare systems rather than large-scale hospitals. These smaller healthcare systems are less likely to report to large databases such as SEER.

More investigations must be done to evaluate the underlying factors contributing to Oklahoma's experience relative to other states. These factors may include the higher prevalence rates of diabetes, hypertension, obesity, and smoking among AI/ANs [17]. A surveillance study of health behaviors performed in 2008 found that AI/ANs were also more likely to be obese and to be current smokers than Whites, and although smoking prevalence has decreased among AI/ANs, these populations still experience a higher prevalence than the general population [18]. Additionally, the question should be asked if barriers to care, such as geographical distance or access to specialty providers, exist in locations where AI/ANs in Oklahoma reside. Espey et al. reported that AI/ANs had a higher poverty rate, lower healthcare coverage, and lower rates of regular source of medical care than Whites [19]. Aside from social or economic factors, the onset of kidney cancer (as with other urologic cancers) is known to be linked to identifiable genomic mutations that arise sporadically or in familial syndromes [20], and an understanding of the pathogenetic prevalence of these specific mutations in AI/AN populations is needed.

Other states with large AI/AN populations have developed innovative ways to combat cancer-related outcome disparities in their localities. For example, South Dakota's *Walking Forward Program* uses a multifaceted approach that combines identifying barriers to care, enhancing navigation through the healthcare system, strengthening treatment delivery while minimizing toxicity, and acquiring molecular data to individualize therapeutics [21]. For kidney cancer that is generally curable when identified early, similar techniques could be employed in Oklahoma to develop risk-stratified enhanced screening programs for early diagnosis in AI/ANs.

There are limitations to consider in this study. Although OCCR is a high-quality database, not all AI/ANs are part of registered tribes nor do they always utilize care provided through Oklahoma's Indian Health Service. Despite the fact that OCCR is regularly linked with IHS race data, misclassification based on race could still be present if certain AI/ANs report themselves as White or do not use the IHS system. Although reporting vital records to the OCCR is required by statute, there still could be missing cases not captured by the database. Finally, these data do not represent the IHS user population, and therefore, this report should not be viewed as reflecting the quality of IHS services.

## 5. Conclusions

AI/ANs in Oklahoma have two times the incidence and mortality rate of kidney cancer relative to Whites. Compared to the U.S. as a whole, both AI/ANs and Whites in Oklahoma fare worse in their susceptibility to acquiring and dying from kidney cancer. These data can be used to develop risk-stratified early screening programs in the context of other multifaceted outreach efforts aimed at minimizing the negative impacts of cancer-related racial disparities.

## Figures and Tables

**(a):** 

Variables		Oklahoma^a^			U.S.^b^		
		AI/AN^c^ (*n* = 1938)	White (*n* = 11,996)	*P* value^d^	AI/AN^c^ (*n* = 6,542)	White (*n* = 809,816)	*P* value^d^
Gender, *n* (%)	Male	1,129 (58)	7,328 (61)	.23	3,925 (60)	506,954 (63)	.13
	Female	809 (42)	4,668 (39)		2,617 (40)	302, 862 (37)	
Age, *n* (%)	<50	400 (21)	1,518 (13)	.18	1,492 (23)	114,083 (14)	.39
	50-69	1,022 (53)	6,007 (50)		3,543 (54)	390,535 (48)	
	>70	501 (26)	4,454 (37)		1,506 (23)	304,225 (38)	
Stage, *n* (%)	Localized	1,114 (58)	6,915 (58)	.50	Not available		
	Regional	2,324 (17)	1,927 (16)				
	Distant	329 (17)	1,983 (17)				
	Unknown	172 (9)	1,171 (10)				
Year, *n* (%)	1997-2003	333 (17)	2,691 (22)	.16	1,003 (15)	160,145 (20)	.03
	2004-2008	406 (21)	2,641 (22)		1,533 (23)	206,904 (26)	
	2009-2013	503 (26)	3,033 (25)		2,078 (32)	233,368 (29)	
	2014-2018	697 (36)	3,631 (30)		2,487 (38)	209,399 (26)	

AI/AN: American Indian/Alaskan Native. ^a^Oklahoma incidence data derived from the Oklahoma State Department of Health (OSDH) web portal, OK2SHARE (https://www.health.state.ok.us/) from 1999 to 2018. ^b^United States incidence data derived from the Center for Disease Control and Prevention Wide-ranging Online Data for Epidemiologic Research (CDC WONDER) database from 1999 to 2017. ^c^AI/AN includes only AI/AN of non-Hispanic origins using IHS racial categories. ^d^Chi-square test. *P* value < .05 statistically significant.

**(b):** 

Variables		Oklahoma^a^			U.S.^b^		
		AI/AN^c^ (*n* = 408)	White (*n* = 2876)	*P* value^d^	AI/AN^c^ (*n* = 1507)	White (*n* = 213,033)	*P* value^d^
Gender, *n* (%)	Male	268 (66)	1,834 (64)	.39	979 (65)	135,699 (64)	.11
	Female	140 (34)	1,042 (36)		528 (35)	77,334 (36)	
Age, *n* (%)	<45	22 (5.0)	48 (2.0)	.21	68 (5.0)	5,271 (3.0)	.46
	45-64	165 (40)	950 (33)		597 (40)	61,202 (29)	
	>65	215 (53)	1,864 (65)		856 (57)	146,584 (69)	
Year, *n* (%)	1999-2003	108 (26)	767 (27)	.33	351 (23)	52,059 (24)	<.01
	2004-2008	109 (27)	817 (28)		397 (26)	54,690 (27)	
	2009-2013	130 (32)	949 (33)		418 (28)	58,103 (27)	
	2014-2015	49 (12)	343 (12)		375 (25)	48,181 (23)	

AI/AN: American Indian/Alaskan Native. ^a^Oklahoma mortality data derived from the Oklahoma State Department of Health (OSDH) web portal, OK2SHARE (https://www.health.state.ok.us/) from 1999 to 2015. ^b^United States mortality data derived from the Center for Disease Control and Prevention Wide-ranging Online Data for Epidemiologic Research (CDC WONDER) database from 1999 to 2017. ^c^AI/AN includes only AI/AN of non-Hispanic origins using IHS racial categories. ^d^Chi-square test. *P* value < .05 statistically significant.

**(a):** 

	AI/AN^a^			White			Rate ratio		
	Overall^d^ (95% CI)	Male^d^ (95% CI)	Female^d^ (95% CI)	Overall^d^ (95% CI)	Male^d^ (95% CI)	Female^d^ (95% CI)	Overall (95% CI)	Male (95% CI)	Female (95% CI)
OK^b^	32.3 (31.1, 33.5)	41.0 (39.0, 43.0)	25.0 (23.5, 26.5)	15.8 (15.5, 16.1)	20.9 (20.4, 21.4)	11.5 (11.1, 11.9)	2.04 (1.96, 2.13)	2.17 (2.03, 2.33)	1.96 (1.86, 2.07)
U.S.^c,e^	14.0 (13.7, 14.3)	18.1 (17.6, 18.6)	10.5 (10.2, 10.8)	15.7 (15.7, 15.7)	21.2 (21.1, 21.3)	11.0 (11.0, 11.0)	0.89 (0.87, 0.91)	0.95 (0.92, 0.99)	0.85 (0.83, 0.88)

AI/AN: American Indian/Alaskan Native. ^a^AI/AN includes only AI/AN of non-Hispanic origins using IHS racial categories. ^b^Oklahoma incidence data derived from the Oklahoma State Department of Health (OSDH) web portal, OK2SHARE (https://www.health.state.ok.us/) from 1999 to 2018. ^c^United States incidence data derived from the Center for Disease Control and Prevention Wide-ranging Online Data for Epidemiologic Research (CDC WONDER) database from 1999 to 2017. ^d^Incidence rates are per 100,000 and are age-adjusted based on the 2000 U.S. standard population. ^e^United States incidence rates excluding AI/ANs and Whites from Oklahoma.

**(b):** 

	AI/AN^a^			White			Rate ratio		
	Overall^d^ (95% CI)	Male^d^ (95% CI)	Female^d^ (95% CI)	Overall^d^ (95% CI)	Male^d^ (95% CI)	Female^d^ (95% CI)	Overall (95% CI)	Male (95% CI)	Female (95% CI)
OK^b^	9.7 (8.9-10.5)	13.8 (12.5-15.1)	6.3 (5.4-7.2)	4.9 (4.7-5.1)	7.0 (6.7-7.3)	3.2 (3.0-3.4)	1.98 (1.81-2.17)	1.97 (1.77-2.19)	1.97 (1.68-2.30)
U.S.^c,e^	3.9 (3.8, 4.0)	5.5 (5.2, 5.8)	2.6 (2.4, 2.8)	4.0 (4.0, 4.0)	5.9 (5.9-5.9)	2.6 (2.6-2.6)	0.98 (0.94, 1.01)	0.93 (0.89, 0.98)	1.00 (0.94, 1.07)

AI/AN: American Indian/Alaskan Native. ^a^AI/AN includes only AI/AN of non-Hispanic origins using IHS racial categories. ^b^Oklahoma mortality data derived from the Oklahoma State Department of Health (OSDH) web portal, OK2SHARE (https://www.health.state.ok.us/) from 1999 to 2015. ^c^United States mortality data derived from the Center for Disease Control and Prevention Wide-ranging Online Data for Epidemiologic Research (CDC WONDER) database from 1999 to 2017. ^d^Mortality rates are per 100,000 and are age-adjusted based on the 2000 U.S. standard population. ^e^United States mortality rates excluding AI/ANs and Whites from Oklahoma.

## Data Availability

The Oklahoma data that support the findings of this study are openly available in a web portal at https://www.health.state.ok.us. The national data that support the findings of this study are openly available in a web portal at https://wonder.cdc.gov.

## Abbreviations

AI/AN: American Indian/Alaskan Natives
AAIR: Age-adjusted incidence rate
AAMR: Age-adjusted mortality rate
APC: Annual percent change
IHS: Indian Health Service
OCCR: Oklahoma Central Cancer Registry.
